# Increased rates of Guillain-Barré syndrome associated with Zika virus outbreak in the Salvador metropolitan area, Brazil

**DOI:** 10.1371/journal.pntd.0005869

**Published:** 2017-08-30

**Authors:** Ashley R. Styczynski, Juliane M. A. S. Malta, Elisabeth R. Krow-Lucal, Jadher Percio, Martha E. Nóbrega, Alexander Vargas, Tatiana M. Lanzieri, Priscila L. Leite, J. Erin Staples, Marc X. Fischer, Ann M. Powers, Gwong-Jen J. Chang, P. L. Burns, Erin M. Borland, Jeremy P. Ledermann, Eric C. Mossel, Lawrence B. Schonberger, Ermias B. Belay, Jorge L. Salinas, Roberto D. Badaro, James J. Sejvar, Giovanini E. Coelho

**Affiliations:** 1 National Center for Emerging Zoonotic and Infectious Diseases, Division of High-Consequence Pathogens and Pathology, Centers for Disease Control and Prevention, Atlanta, Georgia, United States of America; 2 Program for Control and Prevention of Malaria and Diseases Transmitted by Aedes, Brazil Ministry of Health, Brasília, Federal District, Brazil; 3 National Center for Emerging Zoonotic and Infectious Diseases, Division of Vector-Borne Diseases, Centers for Disease Control and Prevention, Fort Collins, Colorado, United States of America; 4 Department of Communicable Disease Surveillance, Brazil Ministry of Health, Brasília, Federal District, Brazil; 5 National Center for Immunization and Respiratory Diseases, Division of Viral Diseases, Centers for Disease Control and Prevention, Atlanta, Georgia, United States of America; 6 National Center for HIV/AIDS, Viral Hepatitis, STD, and TB Prevention, Division of Tuberculosis Elimination, Centers for Disease Control and Prevention, Atlanta, Georgia, United States of America; 7 Federal University of Bahia, Complexo Hospitalar Edgard Santos, Salvador, Bahia, Brazil; Fundacao Oswaldo Cruz, BRAZIL

## Abstract

In mid-2015, Salvador, Brazil, reported an outbreak of Guillain-Barré syndrome (GBS), coinciding with the introduction and spread of Zika virus (ZIKV). We found that GBS incidence during April–July 2015 among those ≥12 years of age was 5.6 cases/100,000 population/year and increased markedly with increasing age to 14.7 among those ≥60 years of age. We conducted interviews with 41 case-patients and 85 neighborhood controls and found no differences in demographics or exposures prior to GBS-symptom onset. A higher proportion of case-patients (83%) compared to controls (21%) reported an antecedent illness (OR 18.1, CI 6.9–47.5), most commonly characterized by rash, headache, fever, and myalgias, within a median of 8 days prior to GBS onset. Our investigation confirmed an outbreak of GBS, particularly in older adults, that was strongly associated with Zika-like illness and geo-temporally associated with ZIKV transmission, suggesting that ZIKV may result in severe neurologic complications.

## Author summary

Shortly following the introduction of Zika virus (ZIKV), a type of flavivirus transmitted by mosquitoes, into Brazil in early 2015, the Brazil Ministry of Health began receiving increased reports of a paralyzing condition known as Guillain-Barré syndrome (GBS). The areas with the greatest number of GBS cases appeared to correlate geographically and temporally with the areas reporting the highest rate of ZIKV infections. This association had been previously observed during a ZIKV outbreak in French Polynesia, however, this had not been systematically examined in a case-control investigation for the ZIKV outbreak in South America. In this investigation, the authors found that the occurrence of GBS in the affected population was nearly four times higher than would be expected, and the risk for GBS was particularly elevated among older adults. GBS was associated with ZIKV-like symptoms and with a combination of ZIKV-like symptoms plus laboratory evidence of a recent flavivirus infection. Taken together, these findings provide strong support for and greater understanding of the link between ZIKV and GBS.

## Introduction

Guillain-Barré syndrome (GBS) is a peripheral polyneuropathy characterized by acute onset of bilateral, symmetric limb weakness with decreased or absent deep-tendon reflexes. GBS is a progressive illness with clinical nadir occurring generally within 2–4 weeks. The underlying disease mechanism by which GBS develops is thought to be related to an aberrant immune response following an infection or other immune stimulation [1]. The most common known inciting infection is *Campylobacter jejuni*, though sporadic cases of GBS have been described temporally following a myriad of other viral, bacterial, and parasitic infections [2]. Several emerging and re-emerging arboviruses, including dengue, chikungunya, West Nile, and Zika viruses, have been associated with isolated cases of GBS [3–6]. The onset of GBS symptoms typically manifests within 6–8 weeks, and particularly the following 10–14 days, after exposure [7]. Although clinical outcomes are generally favorable, approximately 20–30% of cases may develop autonomic disturbances and/or neuromuscular respiratory failure, which are the most common causes of death in GBS [8]. Reported mortality rates range from 3–7% in North America and Europe to 13% in parts of Asia [8, 9].

Zika virus (ZIKV), a flavivirus primarily transmitted by *Aedes* spp. mosquitoes [10–12], was originally identified in Uganda in 1947 [13]. Historically, it has been associated with sporadic cases of rash illness in Africa and Southeast Asia [14–17], though outbreaks of ZIKV began to emerge in the Western Pacific region during the late 2000s. In 2014, an outbreak of ZIKV in French Polynesia was followed by increased reports of GBS. An investigation into the GBS outbreak provided evidence for a possible etiologic association between ZIKV and the cluster of GBS cases [18]. Subsequent reports have further supported this association of ZIKV with severe neurologic sequelae such as GBS and congenital malformations [6, 18–21].

In April 2015, ZIKV was first identified in Brazil, causing an outbreak of exanthematous illness centered in the northeast region [22]. Subsequent to the ZIKV outbreak, clustering of GBS diagnoses was noted in mid-2015 in northeastern Brazil [23–25]. However, to identify risk factors and potential infectious pathogens associated with the reported increase in GBS cases, we performed a case-control investigation to evaluate the relationship between ZIKV and increased reports of suspected GBS. In particular, we sought to establish an etiology for the outbreak of GBS through a case-control investigation using information collected through interviews, and evaluate the relationship of GBS to arboviral infections in the population.

## Materials and methods

### Case ascertainment

We conducted our investigation in the Salvador metropolitan area during January 16 –February 5, 2016. We identified suspected GBS case-patients reported by physicians and hospitals to the Center for Information and Epidemiologic Surveillance of Bahia (Centro de Informações Estratégicas em Vigilância em Saúde [CIEVS]) with onset of neurologic symptoms during January 1– August 31, 2015. To determine compatibility with a GBS diagnosis, we performed medical record reviews to ascertain characteristics of the clinical illness and diagnostic testing, including cerebrospinal fluid, neuroimaging, and electrodiagnostic test results. Suspected GBS case-patients were classified according to diagnostic certainty of the Brighton Collaboration Criteria case definitions for GBS [26]. Case-patients meeting levels 1–3 of diagnostic certainty, and who were at least 12 years of age at time of interview, were classified as confirmed GBS and eligible for enrollment in the case-control investigation.

### Arboviral surveillance

We obtained numbers of suspected ZIKV infections in Salvador during January 1 –August 31, 2015, from CIEVS. We also obtained incidence for suspected and confirmed dengue and chikungunya infections from routine surveillance through the National Notifiable Disease Information System (Sistema de Informação de Agravo de Notificação). We juxtaposed these data to evaluate temporal relationships between dengue, chikungunya, and ZIKV infections compared with confirmed GBS cases.

### Investigation site, design, and participants

For each GBS case-patient, we selected two neighborhood controls from the same general age grouping (12–19, 20–39, 40–59, 60+) as the case-patients. We did this to ensure a relatively equal age distribution between case-patients and controls given that age is a known risk factor for GBS [27]. To identify controls, we flipped a coin to determine the direction of travel from the case-patient’s house, and we used a random number generator to determine how many properties (1–20) to skip to choose the first house. We continued to move in the same direction until finding the first control, and we repeated the random number selection to find the second control, maintaining the direction of travel.

We interviewed all available case-patients and controls to obtain information about demographics, risk factors, and exposures (S1 Table) in the 2 months prior to onset of neurological symptoms of the GBS case-patients. We considered case-patients and controls as having suspected ZIKV disease if they had self-reported symptoms of rash with at least two other ZIKV-like symptoms: fever, conjunctivitis, arthralgia, myalgia, and peri-articular edema [28]. At the time of interview, to determine intermediate-term functional outcomes, we assessed for residual motor deficits using the Hughes GBS Disability Scale [29]. Following the interviews, serum samples were collected from case-patients and controls.

### Laboratory analysis

We tested serum samples by capture enzyme-linked immunosorbent assay for IgM antibodies against ZIKV (MAC-ELISA) and dengue viruses serotypes 1–4 (DENV Detect IgM Capture ELISA, InBios, Inc., Seattle, WA) [30]. We determined neutralizing antibody titers against ZIKV and dengue serotypes 1 and 2 using a 90% cutoff value for plaque-reduction neutralization tests (PRNT_90_) [31]. We defined a recent flavivirus infection as a positive or equivocal IgM test result for ZIKV or dengue. We discriminated between ZIKV and dengue infections if only one PRNT was positive (Table 1) [32].

**Table 1:** Interpretations of antibody testing and sample classification.

PRNT*	Zika or dengue IgM	
	At least one positive (or equivocal)	Both negative
ZIKV ≥ 10, dengue < 10	Recent ZIKV infection	Prior ZIKV infection
ZIKV <10, dengue ≥ 10	Recent dengue infection	Prior dengue infection
ZIKV ≥ 10, dengue ≥ 10	Recent flavivirus infection	Prior flavivirus infection
ZIKV < 10, dengue < 10	No prior ZIKV or dengue exposure	No prior ZIKV or dengue exposure

*PRNT = Plaque-reduction neutralization test

### Statistical analysis

We estimated the incidence of GBS using 2015 population estimates by the Brazilian Institute of Geography and Statistics (Instituto Brasileiro de Geografia e Estatística) [33]. To determine a possible association between GBS and a preceding ZIKV infection, we estimated that 37 case-patients and 74 controls would provide a power of 80% to detect a difference of 30% in ZIKV prevalence, with an alpha level of 5%. We performed logistic regression to calculate odds ratios and 95% confidence intervals for the association of GBS and demographics, known GBS risk factors, antecedent illness, and dengue and ZIKV infections. We also performed sensitivity analyses on combinations of symptoms and laboratory findings to evaluate consistency of results.

### Ethics statement

The human subjects review board at CDC and the Brazil Ministry of Health approved the investigation and determined it to be part of a public health response and not research [National Council of Ethics in Research (Conselho Nacional de Ética em Pesquisa) approval number 1.391.200]. All adult subjects provided informed written consent prior to interview participation and collection of specimens, and a parent or guardian of any child participant (under 18 years old) provided informed consent on their behalf.

## Results

During January 1 –August 31, 2015, 77 suspected GBS case-patients within the Salvador metropolitan area were reported to CIEVS. We reviewed all available medical records, and 50 (65%) patients had sufficient information to be classified as levels 1–3 of the Brighton Collaboration criteria for GBS: 7 (9%) met level 1 of diagnostic certainty, 43 (56%) met level 2, and none met level 3. Of the remaining 27 individuals, 24 (31%) did not have enough information for classification as GBS or had alternative diagnoses. The remaining 3 (4%) were excluded because of factors such as age below enrollment criterion or inaccurate address.

**Fig 1:**
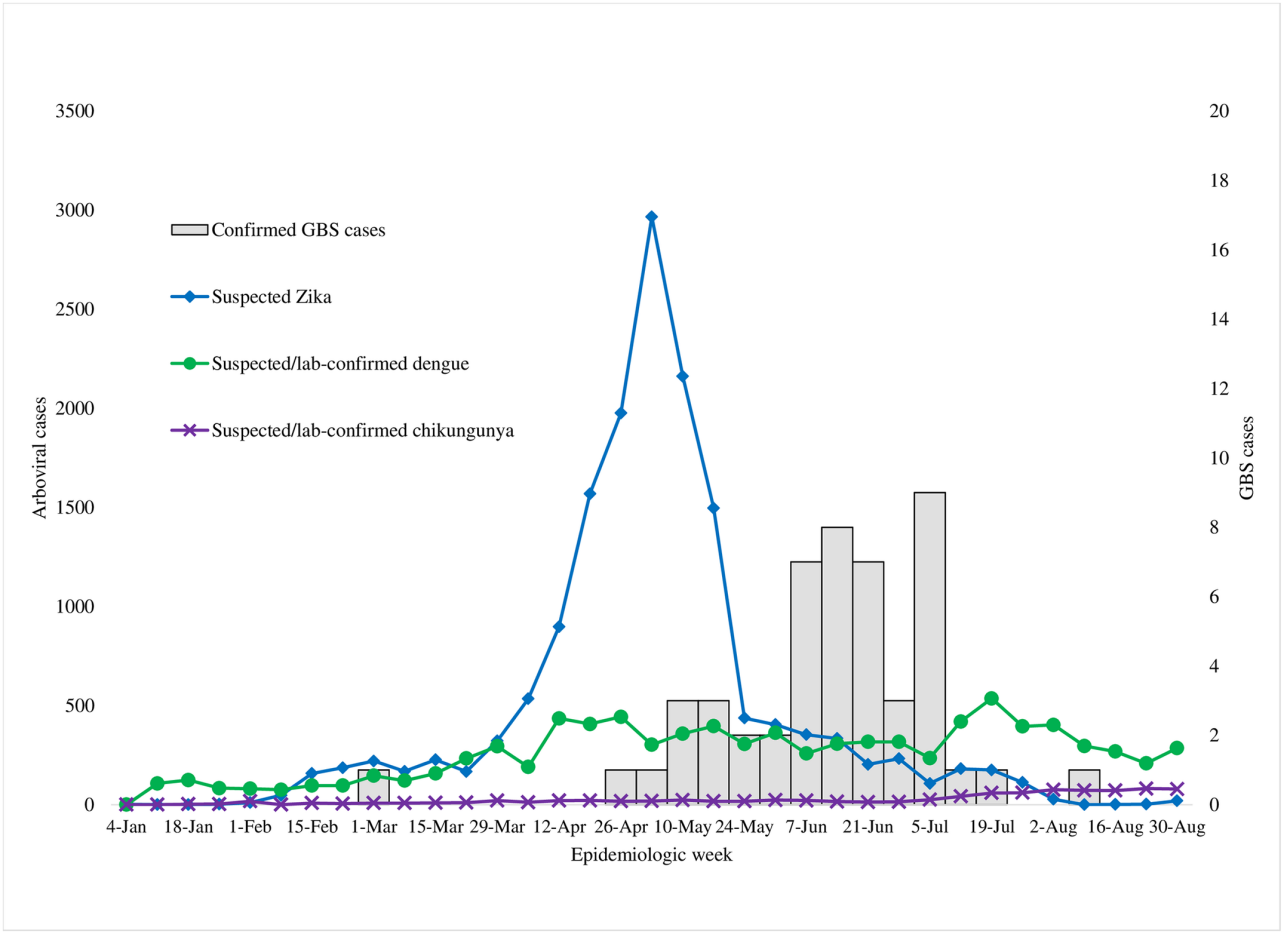
Confirmed GBS and reported Zika, dengue, and chikungunya Cases–Salvador metropolitan area, Brazil 2015. Epidemiologic curve of incident Guillain-Barré syndrome (GBS) cases was juxtaposed with reported symptomatic ZIKV, dengue, and chikungunya infections in the Salvador metropolitan area, Brazil, during January 1–August 31, 2015.

The majority (94%) of Brighton-confirmed case-patients based on medical chart review had neurologic illness onset during epidemiologic weeks 17–29 (April 26 –July 25) (Fig 1). During this period of peak GBS occurrence, the annualized incidence of GBS was 5.6 cases/100,000 population for the Salvador metropolitan area. Annualized age-group-specific incidence increased with age. The incidence was 1.5 cases/100,000 population for the 12–19 years age group, 3.9 for the 20–39 years age group, 7.3 for the 40–59 years age group, and 14.7 among persons ≥60 years of age. The median age of these cases was 47 years (range, 14–79 years). There was no significant difference in incidence between men and women (5.8 versus 5.5, p = 0.37).

Based on medical chart review of the 50 confirmed GBS case-patients, 44 were reported to have had a preceding illness (S2 Table); the median time between antecedent illness and neurologic symptom onset was 8 days (IQR 5–15). Prominent neurologic signs/symptoms of the GBS case-patients included leg and arm weakness, dysphagia, and facial weakness. Median time from onset of neurologic symptoms to nadir was 6 days (IQR 4–9). Nine case-patients had electrodiagnostic studies available for review. Of the available reports, 5 were interpreted as being consistent with the acute motor axonal neuropathy (AMAN) subtype of GBS, and the other 4 demonstrated patterns interpreted as the acute inflammatory demyelinating polyradiculoneuropathy (AIDP) subtype of GBS. All case-patients were hospitalized; 46 (92%) received intravenous immunoglobulin (IVIG), 17 (34%) required ICU-level care, 11 (22%) required mechanical ventilation, and 3 (6%) died (Table 2).

**Table 2:** Clinical characteristics of case-patients with Guillain-Barré syndrome (N = 50) in the Salvador metropolitan area—Brazil, 2015.

	N (%) or median [IQR]
Time between onset of previous illness and onset of neurological symptoms, d	8 [5–15]
Time from onset of symptoms until nadir, days	6 [4–9]
Symptoms at onset	
Lower extremity weakness	20 (40)
Upper extremity weakness	3 (6)
Facial weakness	4 (8)
Lower extremity paresthesias	29 (58)
Upper extremity paresthesias	17 (34)
Facial paresthesias	1 (2)
Dysphagia	1 (2)
Diplopia/ophthalmoplegia	0 (0)
Gait disturbance	5 (10)
Symptoms at any time	
Lower extremity weakness	35 (70)
Upper extremity weakness	35 (70)
Facial weakness	25 (50)
Lower extremity paresthesias	20 (40)
Upper extremity paresthesias	22 (44)
Facial paresthesias	9 (18)
Dysphagia	25 (50)
Diplopia/ophthalmoplegia	3 (6)
Gait disturbance	4 (8)
Cytoalbuminologic dissociation	44 (88)
Level of care	
Hospitalized	50 (100)
Intensive care	17 (34)
Mechanical ventilation	11 (22)
Intravenous immune globulin	46 (92)

The reports of suspected symptomatic ZIKV infections occurred as a prominent clustering during April–June of 2015 while no apparent fluctuations were reported in either dengue or chikungunya infections throughout 2015. The outbreak of GBS peaked approximately 7 weeks after the peak of suspected ZIKV infections (Fig 1).

Of the 47 GBS case-patients who were alive at the time of the investigation, 2 declined participation and we could not locate 4, leaving 41 individuals that we could further evaluate through interview. The median age for these case-patients was 44 years (range 14–78), which was not significantly different from the controls with a median age of 50 (range 13–87). Additional demographics and exposure histories did not differ between case-patients and controls (S1 Table) with the exception that a higher proportion of GBS case-patients compared to controls reported an antecedent illness in the 2-month period prior to neurologic symptom onset of GBS case-patients (Table 3). Symptoms most frequently reported by GBS case-patients included rash, headache, fever, myalgias, and arthralgias.

**Table 3:** Demographics and risk factors of case-patients with Guillain-Barré syndrome and controls enrolled in the case-control investigation, Salvador metropolitan area, Brazil, 2015.

	N (%) or median [IQR]		Odds ratio	95% CI
**Demographics**	Cases, n = 41	Controls, n = 85		
Age, y	44 [32–54]	50 [33–59]	--	
Men	22 (54)	34 (40)	1.74	(0.82–3.68)
Race (self-report)				
White	5 (12)	6 (7)	ref*	
Black	13 (32)	29 (34)	1.86	(0.48–7.21)
Mixed	20 (49)	45 (53)	1.88	(0.51–6.87)
Other	2 (5)	3 (4)	1.25	(0.15–10.70)
≥1 Chronic medical condition	21 (51)	51 (60)	0.70	(0.33–1.48)
**Risk factors** (within 2 months of neurologic symptom onset of case-patients)				
Mosquito exposure	22 (54)	55 (65)	1.35	(0.60–3.05)
Recent vaccination	6 (15)	9 (11)	1.45	(0.48–4.38)
Recent surgery	0 (0)	3 (4)	undef†	
Previous illness	34 (83)	18 (21)	18.08	(6.88–47.49)
**Viral-like syndrome**				
Rash	24 (59)	7 (8)	15.73	(5.83–42.42)
Fever	21 (51)	13 (15)	5.82	(2.48–13.62)
Conjunctivitis	11 (27)	4 (5)	7.43	(2.20–25.12)
Arthralgias	18 (44)	12 (14)	4.76	(2.00–11.34)
Myalgias	20 (49)	11 (13)	6.41	(2.66–15.46)
Headache	23 (56)	14 (16)	6.48	(2.79–15.04)
Periarticular edema	7 (17)	6 (7)	2.71	(0.85–8.67)
**Gastrointestinal syndrome**				
Nausea/vomiting	11 (27)	4 (5)	7.43	(2.20–25.12)
Diarrhea	9 (22)	6 (7)	3.70	(1.22–11.26)
Abdominal pain	4 (10)	5 (6)	1.73	(0.44–6.82)
**Upper respiratory syndrome**				
Rhinorrhea	2 (5)	4 (5)	1.04	(0.18–5.92)
Cough	4 (10)	5 (6)	1.73	(0.44–6.82)
Sore throat	3 (7)	6 (7)	1.04	(0.25–4.38)
**Laboratory findings**	(n = 41)	(n = 84)		
Recent Zika virus infection	0	0	--	
Prior Zika virus infection	0	0	--	
Recent dengue	0	0	--	
Recent flavivirus	18 (44)	32 (38)	1.27	(0.60–2.71)
No evidence of Zika virus infection (PRNT‡ neg)	5 (12)	20 (24)	0.44	(0.15–1.29)
**Case definitions**				
Suspected Zika virus disease	21 (51)	6 (7)	13.83	(4.93–38.78)
Suspected Zika virus disease + lab evidence of recent flavivirus infection	10 (24)	4 (5)	6.45	(1.88–22.10)

* ref = reference category.

^†^ undef = undefined.

^‡^ PRNT = plaque-reduction neutralization test.

At the time of our assessment, all living GBS case-patients were at least 5 months out from neurologic symptom onset (median 220 days, range 160–321 days). Thirty-five case-patients (85%) reported at least minor residual motor deficits; 17 (41%) had substantial residual motor deficits and could not walk without assistance.

Case-patients were found to meet criteria for suspected ZIKV disease significantly more often than controls (Table 3). There were no case-patients or controls who met laboratory criteria for recent or prior ZIKV infection or recent dengue infection. Recent flavivirus infections were equally prevalent between case-patients and controls. However, being a case-patient was significantly associated with evidence of recent flavivirus infection when combined with clinical criteria for suspected ZIKV disease. Only a small number of case-patients and controls had no evidence of prior ZIKV exposure based on negative PRNTs. Nearly all of the samples tested positive for dengue (serotypes 1 and 2) virus-neutralizing antibodies (100% of case-patients versus 96% of controls), leaving only 3 controls with no evidence of prior exposure to ZIKV, dengue, or other flaviviruses.

## Discussion

Our investigation demonstrated a high incidence of GBS geographically and temporally clustered in the setting of an ongoing large outbreak of ZIKV. GBS case-patients in this investigation were more likely than non-GBS controls to report symptoms suggestive of ZIKV illness in the 6–8 weeks prior to neurologic illness onset; in addition, based on both medical record review and self-report, the distribution of onsets of the antecedent illnesses clustered during the 2 weeks before the onset of neurologic symptoms. This temporal relationship supports an etiological association between these two illnesses. Despite this epidemiological evidence, the serologic data could not confirm this relationship. However, the serologic findings from specimens collected an average of 7 months after GBS onset showed a high prevalence of ZIKV-neutralizing antibodies in cases and controls, consistent with the ZIKV epidemic that was recognized in the community during the preceding months [22].

Baseline population estimates of GBS incidence in Brazil are limited but are reported at 0.05–0.6 cases/100,000 people per year, which are substantially lower than expected [34–36]. The incidence of GBS in North America and Europe is 0.81–1.89 with an expected worldwide incidence of 1.1–1.8 cases per 100,000 people per year [27, 37]. Applying a baseline estimate of 1.5 cases per 100,000 people per year results in a 3.7-times increased incidence of GBS for the outbreak period for persons at least 12 years of age.

The characteristics of GBS illness during this outbreak were largely similar to what would be expected for typical GBS disease patterns with some notable exceptions. The high attack rates in the older individuals reflects an unusually steep increase in GBS incidence during the outbreak. The incidence of GBS in this investigation is 10-times higher among the oldest age group compared with the youngest, in contrast to a 2–3-times increase in incidence reported elsewhere for the same population groups [27]. Indicators of GBS severity, such as need for intensive care monitoring and mechanical ventilation, were comparable to other reports of GBS [1]. Overall, the 6% mortality rate was similar to rates in North America and Europe and possibly reflected the high utilization of IVIG and supportive care of the GBS case-patients in this outbreak. Most of the case-patients regained lost function after their GBS illness; however, 41% of case-patients still required assistance with walking 6 months later, which is higher than the 20% that is more commonly expected [38]. Additionally, there was a more rapid progression to nadir than the 2–4 weeks usually observed for GBS, though this was similar to findings in French Polynesia [18, 39, 40].

Few studies exist that characterize the subtypes and electrophysiologic findings of GBS in Brazil [41]. Though there was a limited number of electrophysiologic studies performed for case-patients in this investigation, there appeared to be a relatively equal distribution of AMAN and AIDP subtypes. This contrasts with French Polynesia where AMAN was the predominant subtype and with Puerto Rico and Colombia where AIDP was most frequently reported [18, 20, 21]. Additional investigations are required to define the electrophysiologic features of ZIKV-associated GBS, which could contribute to understanding the underlying pathophysiologic mechanisms.

We noted a 7-week interval between the peaks of reported ZIKV infections and GBS, which is longer than would be expected if ZIKV was biologically associated with GBS. This finding was consistent with data reported elsewhere [25]. However, this differed from individual-level data, in which the median interval between onset of ZIKV-like illness and GBS was 8 days. Several factors may have contributed to this effect. One possibility suggested by Paploski, et al. [25] is that once the community perceived ZIKV infections as benign, persons may have stopped seeking care, artificially foreshortening the epidemiologic peak of ZIKV infections. Alternatively, there may be limitations in the surveillance data for ZIKV infections given that very few case-patients had laboratory confirmation. This is especially notable considering recent reports that more closely align occurrences of ZIKV infections and GBS in Bahia [6].

The preceding illnesses of the case-patients were most prominently characterized by rash, headache, fever, myalgias, and arthralgias—symptoms commonly reported with ZIKV infections [42]. The occurrence of acute illness among the case-patients 8 days prior to onset of GBS provides a biologically plausible argument for a causal association between the acute illness and GBS and has been similarly reported in other studies of Zika-associated GBS [20, 21]. We found that there was also a significant, but much less robust, association for gastrointestinal symptoms, such as nausea/vomiting and diarrhea. Gastrointestinal symptoms have been reported in the setting of other ZIKV outbreaks [20, 43], and it is possible that these symptoms are an under-recognized clinical feature of ZIKV illness, rather than manifestations of *Campylobacter* infection leading to the GBS cases observed in this investigation. However, it is probable that not all of the case-patients had the same antecedent etiology, which is supported by the fact that several of the case-patients did not report symptoms of Zika-like illness or have laboratory evidence of previous ZIKV infection.

The attack rate of ZIKV in northeastern Brazil is unknown. However, if it was as high as reported in French Polynesia, it could limit our ability to detect differences in seropositivity between case-patients and controls. Tests of association cannot discriminate between such high rates of infection without a very large sample size. Notwithstanding, the laboratory data can be used to demonstrate likely ZIKV exposure, which hence could be presumed to represent a relatively recent infection since the virus has only been identified in Brazil since early 2015. Additionally, given this finding of greater ZIKV-like symptoms in the case-patients, even if rates of ZIKV infection are not different between the case-patients and controls, it may indicate that individuals with symptomatic ZIKV infections are more predisposed to the development of GBS. It is not yet understood what factors lead to symptomatic versus asymptomatic infections, though possibilities may include prior infectious exposures causing potentiation, initial infectious viral load, or host immunologic response. The latter theory is supported by this observed correlation with GBS, which is itself a manifestation of an aberrant immune response. Further exploration of the immunopathogenesis of ZIKV may provide insight into the mechanism of Zika-associated GBS.

Because patients for whom GBS case status was not ascertained were not interviewed, we did not systematically collect demographic information on them, so no comparisons could be made with case-patients enrolled in the investigation. The major limitations in antecedent illness analysis are the non-specific nature of the symptoms, possible underreporting of acute illnesses by controls, and recall bias in reporting of symptoms by case-patients. Because the investigation was performed approximately 7 months after the acute illness onset, laboratory findings were limited to serology. Because there is no information on how long ZIKV-specific IgM persists, the finding of a negative IgM result so far out from the initial suspected ZIKV infection is difficult to interpret. Furthermore, the cross-reactivity of dengue and ZIKV antibodies makes accurate discrimination between these pathogens particularly challenging since all samples with ZIKV-specific neutralizing antibodies also had dengue virus-specific neutralizing antibodies [32, 44], though measuring ZIKV-specific neutralizing antibodies several months from the acute infection may be a more specific indicator [45]. Regardless, the laboratory findings more effectively document flavivirus exposure rather than being able to confirm Zika infection or exposure, and other possible infectious triggers cannot be ruled out; in particular, *Campylobacter*-specific surveillance data was not available and could not be retrospectively ascertained. Additionally, ascertainment of neurological impairment was based on retrospective abstraction of medical records, which may be limited by lapses in documentation.

The findings of this investigation, along with similar epidemiologic findings throughout Latin America and previously published data from French Polynesia [6, 18, 20, 21, 25], strongly suggest a ZIKV-associated GBS. The apparent etiological association between ZIKV and GBS and the observed higher ZIKV-related GBS attributable risk in older persons should be substantiated through additional prospective studies of GBS during ZIKV outbreaks. These studies should include molecular confirmation of infection to further define distinctive clinical and laboratory features of such cases. If this etiologic relationship is true, prevention of GBS may be enhanced by minimization of exposure to mosquitoes through personal protection and environmental control methods. It may also be prudent to target public health messaging about GBS to older adult populations during ZIKV outbreaks. Additionally, these findings may inform future preparedness efforts to build GBS-related diagnostic, treatment, and hospital capacity in areas at risk for ZIKV infection.

## Supporting information

S1 Table Associations between antecedent symptoms, exposures, and laboratory findings among case-patients with Guillain-Barré syndrome and controls—Salvador metropolitan area, Brazil, 2015. (DOCX)

S2 Table Antecedent symptoms reported in chart review among 50 confirmed Guillain-Barré syndrome cases—Salvador metropolitan area, Brazil, 2015. (DOCX)
