# Supplementary material for: Increased rates of Guillain-Barré syndrome associated with Zika virus outbreak in the Salvador metropolitan area, Brazil
Source: PLoS Negl Trop Dis. 2017 Aug 30;11(8):e0005869. doi: 10.1371/journal.pntd.0005869 (PMC5595339; doi:10.1371/journal.pntd.0005869)
Supplement: S1 Table — (DOCX) [file pntd.0005869.s001.docx]

**Supplementary Appendix 1**

**Table.** Associations between antecedent symptoms, exposures, and laboratory findings among case-patients with Guillain-Barré syndrome and controls — Salvador metropolitan area, Brazil, 2015

| **Questionnaire item** | **N (%)†** | | **OR 95% CI** | |
| --- | --- | --- | --- | --- |
| **Symptoms reported** | Cases, n=41 | Controls, n=85 |  | |
| Rash | 24 (59) | 7 (8) | 15.73** | [5.83-42.42] |
| Fever | 21 (51) | 13 (15) | 5.82** | [2.48-13.62] |
| Chills | 15 (37) | 11 (13) | 3.88* | [1.58-9.52] |
| Pruritus | 17 (41) | 8 (9) | 6.82** | [2.62-17.76] |
| Nausea/vomiting | 11 (27) | 4 (5) | 7.43** | [2.20-25.12] |
| Diarrhea | 9 (22) | 6 (7) | 3.70* | [1.22-11.26] |
| Myalgia | 20 (49) | 11 (13) | 6.41** | [2.66-15.46] |
| Arthralgia | 18 (44) | 12 (14) | 4.76** | [2.00-11.34] |
| Periarticular edema | 7 (17) | 6 (7) | 2.71 | [0.85-8.67] |
| Conjunctivitis | 11 (27) | 4 (5) | 7.43** | [2.20-25.12] |
| Headache | 23 (56) | 14 (16) | 6.48** | [2.79-15.04] |
| Retroorbital pain | 15 (37) | 7 (8) | 6.43** | [2.36-17.49] |
| Nuchal rigidity | 5 (12%) | 6 (7) | 1.83 | [0.52-6.39] |
| Abdominal pain | 4 (10) | 5 (6) | 1.73 | [0.44-6.82] |
| Cough | 4 (10) | 5 (6) | 1.73 | [0.44-6.82] |
| Rhinorrhea | 2 (5) | 4 (5) | 1.04 | [0.18-5.92] |
| Calf pain | 11 (27) | 7 (8) | 4.09* | [1.45-11.52] |
| Odynophagia | 3 (7) | 6 (7) | 1.04 | [0.25-4.38] |
| Confusion | 2 (5) | 1 (1) | 4.31 | [0.38-48.95] |
| **Food exposures** (weekly) | |  |  |  |
| Beef | 41 (100) | 82 (96) | Undefined |  |
| Chicken | 39 (95) | 84 (99) | 0.23 | [0.02-2.64] |
| Lamb | 9 (22) | 19 (22) | 0.98 | [0.40-2.40] |
| Fish | 33 (80) | 77 (91) | 0.43 | [0.15-1.24] |
| Shellfish | 14 (34) | 44 (52) | 0.48 | [0.22-1.05] |
| Milk | 33 (80) | 75 (88) | 0.55 | [0.20-1.52] |
| Cheese | 28 (68) | 73 (86) | 0.35* | [0.14-0.87] |
| Yogurt | 26 (63) | 56 (66) | 0.90 | [0.41-1.95] |
| Salad | 32 (78) | 75 (88) | 0.47 | [0.18-1.28] |
| **Animal exposures** |  |  |  |  |
| Dogs | 20 (49) | 40 (47) | 1.07 | [0.51-2.26] |
| Cats | 11 (27) | 23 (27) | 0.99 | [0.43-2.29] |
| Rats | 12 (29) | 24 (28) | 1.05 | [0.46-2.39] |
| Birds | 12 (29) | 25 (29) | 0.99 | [0.44-2.25] |
| Lizards/turtles | 5 (12) | 7 (8) | 1.55 | [0.46-5.21] |
| Goats | 0 | 0 | Undefined |  |
| Sheep | 0 | 0 | Undefined |  |
| Cows | 0 | 1 (1) | Undefined |  |
| Chickens | 4 (10) | 4 (5) | 2.19 | [0.52-9.24] |
| Pigs | 0 | 1 (1) | Undefined |  |
| **Environmental exposures** |  |  |  |  |
| Drinking tap water  Never  Rarely  Sometimes  Almost always | 6 (15)  5 (12)  8 (20)  22 (54) | 14 (16)  8 (9)  7 (8)  56 (66) | ref  1.46  2.67  0.92 | [0.34-6.35]  [0.66-10.75]  [0.31-2.69] |
| Tap water boiled/treated (yes) | 13 (36), n=36 | 31 (42), n=73 | 0.77 | [0.34-1.75] |
| Consuming untreated water (well, river/stream/pond)  Never  Rarely  Sometimes  Almost always | 37 (90)  2 (5)  0  2 (5) | 71 (85), n=84  7 (8)  5 (6)  1 (1) | ref  0.55  Undefined  3.84 | [0.11-2.77]  [0.34-43.73] |
| Other water boiled/treated (yes) | 1 (7), n=14 | 3 (9), n=35 | 0.82 | [0.08-8.63] |
| Walking barefoot outside  Never  Rarely  Sometimes  Almost always | 34 (83)  1 (2)  3 (7)  3 (7) | 56 (66)  21 (25)  6 (7)  2 (2) | ref  0.08*  0.82  2.47 | [0.01-0.61]  [0.19-3.51]  [0.39-15.54] |
| Swimming in rivers or freshwater  Never  Rarely  Sometimes  Frequently | 34 (83)  4 (10)  3 (7)  0 | 63 (75), n=84  14 (17)  3 (4)  4 (5) | ref  0.53  1.85  Undefined | [0.16-1.74]  [0.36-9.69] |
| Hours spent outdoors  1–4 hours  5–8 hours  >8 hours | 16 (46), n=35  9 (26)  10 (29) | 32 (44), n=72  17 (24)  23 (32) | ref  1.06  0.87 | [0.39-2.90]  [0.34-2.26] |
| Mosquito bite (yes) | 22 (61), n=36 | 55 (68), n=81 | 0.74 | [0.33-1.68] |
| Insect repellent  Never  Rarely  Sometimes  Almost always | 30 (73)  6 (15)  3 (7)  2 (5) | 58 (69), n=84  17 (20)  6 (7)  3 (4) | ref  0.68  0.97  1.29 | [0.24-1.91]  [0.23-4.14]  [0.20-8.14] |
| Open windows  Never  During the day  At night  Always | 0  23 (56)  1 (2)  17 (41) | 3 (4)  54 (64)  0  28 (33) | Undefined  ref  Undefined  1.43 | [0.66-3.10] |
| Window screens  None  Some windows  All | 37 (90)  2 (5)  2 (5) | 79 (93)  3 (4)  3 (4) | ref  1.42  1.42 | [0.23-8.89]  [0.23-8.89] |
| Air conditioner (yes) | 7 (17) | 6 (7) | 2.71 | [0.85-8.67] |
| Fan (yes) | 40 (98) | 76 (89) | 4.74 | [0.58-38.73] |
| Presence of standing water  Never  Every other week  Once a week  2–3 times/week  Daily | 21 (51)  1 (2)  1 (2)  2 (5)  16 (39) | 38 (45), n=84  2 (2)  1 (1)  5 (6)  38 (45) | ref  0.91  1.81  0.72  0.76 | [0.08-10.58]  [0.11-30.44]  [0.13-4.06]  [0.35-1.68] |
| Slaughtered animals (yes) | 3 (7) | 7 (8) | 0.87 | [0.21-3.55] |
| Handled dead animals (yes) | 2 (5) | 10 (12) | 0.39 | [0.08-1.84] |
| Recent illness of family member (yes) | 13 (32) | 22 (28), n=79 | 1.20 | [0.53-2.74] |
| **Laboratory findings** | Cases (n=41) | Controls (n=84) |  |  |
| ZIKV IgM positive/equivocal | 17 (41) | 30 (36) | 1.28 | [0.59-2.74] |
| Dengue IgM positive/equivocal | 4 (10) | 5 (6) | 1.71 | [0.43-6.73] |
| ZIKV PRNT positive (≥10) | 36 (88) | 64 (76) | 2.25 | [0.78-6.51] |
| Dengue PRNT positive (≥10) | 41 (100) | 81 (96) | Undefined |  |
| Recent ZIKV infection | 0 | 0 | Undefined |  |
| Recent ZIKV/flavivirus (non-dengue) infection | 18 (44) | 32 (38) | 1.27 | [0.60-2.71] |

**†** For cases, n=41, and for controls, n=85, unless otherwise specified; ref = reference category

**Significant at p<0.001

*Significant at p<0.05
