# Supplementary material for: Increased rates of Guillain-Barré syndrome associated with Zika virus outbreak in the Salvador metropolitan area, Brazil
Source: PLoS Negl Trop Dis. 2017 Aug 30;11(8):e0005869. doi: 10.1371/journal.pntd.0005869 (PMC5595339; doi:10.1371/journal.pntd.0005869)
Supplement: S2 Table — (DOCX) [file pntd.0005869.s002.docx]

**Supplementary Appendix 2**

**Table.** Antecedent symptoms reported in chart review among 50 confirmed Guillain-Barré syndrome cases — Salvador metropolitan area, Brazil, 2015

| Antecedent symptom | N (%) |
| --- | --- |
| Rash | 21 (42) |
| Arthralgia | 13 (26) |
| Myalgia | 17 (34) |
| Headache | 11 (22) |
| Cough | 0 |
| Abdominal pain | 1 (2) |
| Nausea/vomiting | 4 (8) |
| Conjunctivitis | 3 (6) |
| Chills | 0 |
| Diarrhea | 8 (16) |
| Rhinorrhea | 0 |
| Retroorbital pain | 1 (2) |
| Nuchal rigidity | 0 |
| Confusion | 0 |
| Odynophagia | 0 |
| Calf pain | 0 |
| Total | 50 (100) |
